# Exploration of Shared Themes Between Food Security and Internet of Things Research Through Literature-Based Discovery

**DOI:** 10.3389/frma.2021.652285

**Published:** 2021-05-13

**Authors:** Cristian Mejia, Yuya Kajikawa

**Affiliations:** ^1^Graduate School of Environment and Society, Tokyo Institute of Technology, Tokyo, Japan; ^2^Institute for Future Initiatives, The University of Tokyo, Tokyo, Japan

**Keywords:** literature-based discovery, citation networks, text mining, food security, poverty alleviation, SDGs, Internet of Things

## Abstract

This paper applied a literature-based discovery methodology utilizing citation networks and text mining in order to extract and represent shared terminologies found in disjoint academic literature on food security and the Internet of Things. The topic of food security includes research on improvements in nutrition, sustainable agriculture, and a plurality of other social challenges, while the Internet of Things refers to a collection of technologies from which solutions can be drawn. Academic articles on both topics were classified into subclusters, and their text contents were compared against each other to find shared terms. These terms formed a network from which clusters of related keywords could be identified, potentially easing the exploration of common themes. Thirteen transversal themes, including blockchain, healthcare, and air quality, were found. This method can be applied by policymakers and other stakeholders to understand how a given technology could contribute to solving a pressing social issue.

## Introduction

Literature-based discovery (LBD) refers to text mining methodologies aimed at connecting disjoint literature by finding intermediary terms or concepts. Bridging terms help experts and practitioners derive hypotheses that can be tested in the research labs. As such, LBD is a tool for the systematic creation of hypotheses, potentially accelerating the discovery of solutions to known problems (Kostoff, 2006). For instance, Swanson (Swanson, 1986) established a connection between Raynaud's syndrome and fish oil as a potential treatment by mining academic articles on both topics and finding linking terms, such as blood viscosity. This treatment was validated through clinical trials.

While the application of text mining methods has extended across most fields of science, LBD methods continued to be focused on biomedical research due to their valuable contributions in finding linkages between diseases and potential treatments (Coeckelbergh et al., 2016; Gopalakrishnan et al., 2019). Most LBD methodologies fall within two models: open or closed discovery. In open discovery, researchers start with a seed term or topic, with subsequent steps to find related terms. This process can result in an ever-expanding list of related terms from where unexpected but valuable connections can be found. In closed discovery, researchers have an idea regarding two topics for comparison. The corpus of knowledge representing both topics is usually referred to as literature A and literature C, and a methodology is applied to find connecting terms, usually called B-terms. These B-terms are the output of the method and presented to experts who attempt to draw hypotheses on the utility of terms (Henry and McInnes, 2017).

In most cases, the process of systematic discovery of B-terms follows a generic framework composed of data acquisition or the selection of data sources and data types, the discovery process, output representation process or visualization, and validation (Thilakaratne et al., 2019a). Each has a possibility of applying a variety of methods depending on the nature of the study and data type under analysis. The discovery process has been performed by applying computation techniques, such as fuzzy logic, topic models, and clustering analysis (Thilakaratne et al., 2019b). Most of them establish associations by exploring keywords or their context within sentences in the text (Cameron et al., 2015). However, combination methods utilizing bibliographic information of academic articles and semantic context are more accurate when predicting future associations between a pair of terms (Sebastian et al., 2017).

A seemingly common trend in biomedical LBD is the reliance on medical thesauri, ontologies, and controlled vocabularies, such as the Unified Medical Language System (UMLS) (Weeber et al., 2001) or the Medline Subject Heading (MESH) (Baker, 2010). During the input stage, only keywords matching those found in the thesaurus are used in the discovery process, with the output list of B-terms including concepts familiar to biomedical fields. This eases the inference process of establishing hypotheses, as meaningless keywords are sorted by default. The advantage of controlled vocabularies is missing in non-biomedical fields, which may hamper the adoption of LBD methods, as more effort is required to remove unnecessary keywords from output B-terms, establish hierarchies and classifications, and validate the concepts.

Few studies have explored the application of LBD beyond biomedical research (Hui and Lau, 1997). Some early examples include the application of LBD to find influences among poetry writers (Cory, 1997), study the spread of genetic algorithms in the World Wide Web (Gordon et al., 2002), and establish connections among persons, places, and other entities in counter-terrorism databases (Jha and Jin, 2016). Most other applications were in the fields of innovation and technology management. For instance, Kostoff et al. (2008) explored the applicability of LBD in finding technologies related to water purification, while Huang et al. (2012) used a similar approach focused on agricultural economics. This involved the integration of text mining with network analysis in the works of technology management scholars. For instance, Fujita (2012) applied a mixed method of citation networks and text mining to find intermediary concepts between sustainability science and the field of complex networks.

In addition, there is a trend of attempting to bridge technological concepts with social issues. Ittipanuvat et al. (2014) explored the linkage between robotics and gerontology. This study compared and analyzed over 11,000 articles on robotics and 22,000 on gerontology, making it one of the first to deal with a relatively large volume of data. This study helped identify ten specific robotic technologies (e.g., laparoscopic surgery) that potentially address 13 specific problems among the elderly (e.g., prostate cancer). Likewise, Takano and Kajikawa (2018) linked the Internet of Things (IoT) to the topics of water, energy, health, agriculture, and biodiversity. In previous cases, the researchers successfully identified relationship patterns in literature on technological solutions and social issues at the cost of laborious validation by experts, given that LBD methods shown in those articles signaled similarities between pairs of subtopics, and the experts noted the logical connection between them.

In contrast to the methods used in previous research, this study aimed to reduce the burden of exploring a list of B-terms between two disjoint literatures by grouping them into themes and presenting them graphically. This study took advantage of the analysis of gaps to find undiscovered public knowledge, including logical associations, solutions, and applications already in place in unexpected research fields or overlooked due to information overflow (Swanson, 1986; Smalheiser, 2018).

This method is illustrated by bridging research on food security and the Internet of Things (IoT). The first corresponds to the second United Nations (UN) Sustainable Development Goal (SDG), that is, to “end hunger, achieve food security and improved nutrition, and promote sustainable agriculture” (United Nations, 2015). The latter is a term for several technologies expected to enhance the quality of life and exert great economic impact (Manyika et al., 2015). Food security literature represents a collection of social issues, while research on IoT provides a collection of solutions. This method should shed light on common themes between research areas where synergies could be possible. The methodology applied in this study was first presented in the First International Workshop on Literature-Based Discovery in the Pacific-Asia Conference on Knowledge Discovery and Data Mining (Mejia and Kajikawa, 2020). This paper provides a detailed explanation of each step, improves the implementation and reproducibility of the methods, and targets a new case study.

## Data and Methods

**Figure 1:**
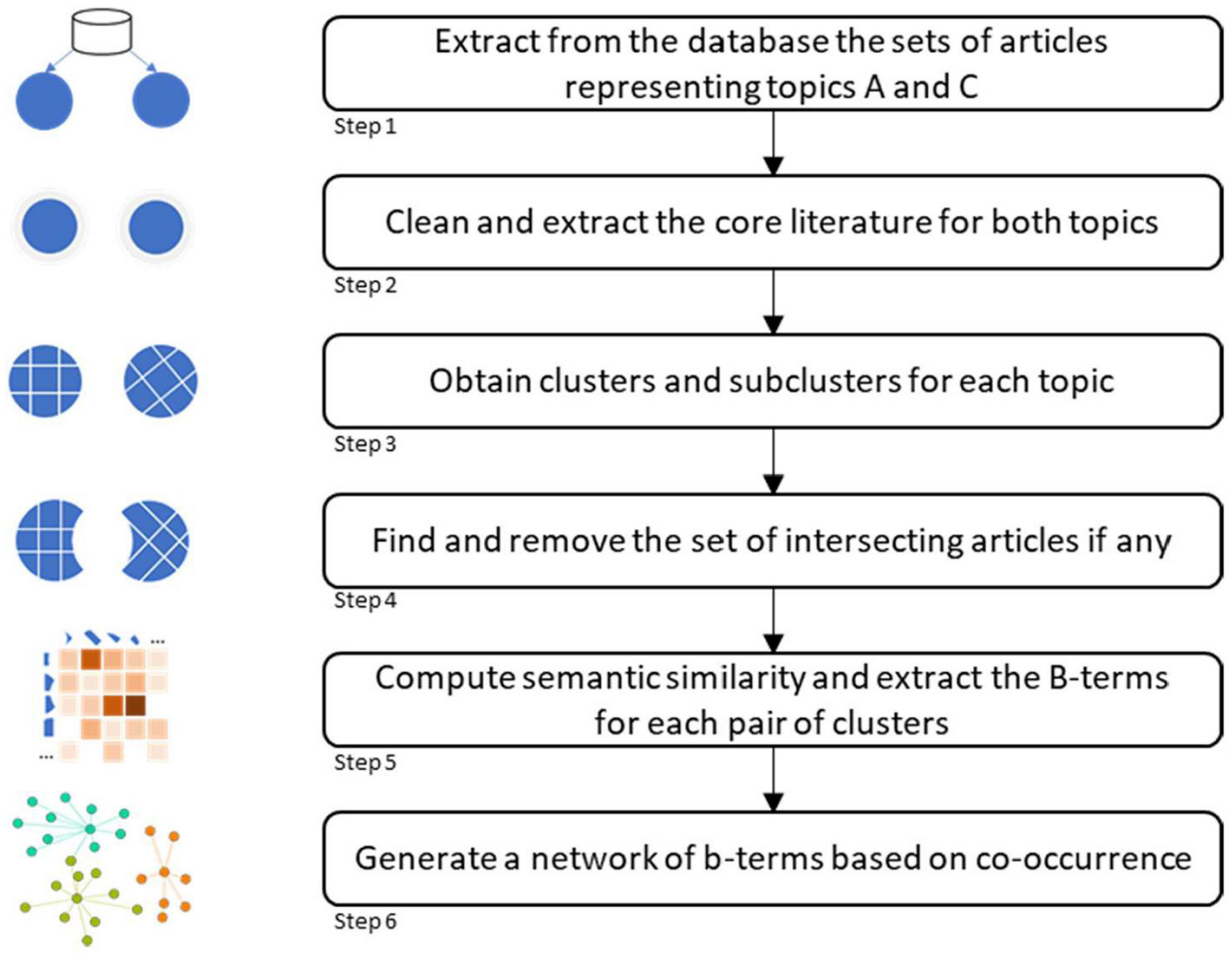
Overview of the methodology.

An overview of the methodological approach is shown in Figure 1. It consists of six steps, including data acquisition and methodological approach. Each step is explained in the following subsections.

### Step 1

First, literature related to topics of food security and IoT was compiled. Bibliographic data from the Web of Science Core Collection, which covers articles from the sciences, social sciences, arts, and humanities, was retrieved. To obtain the articles, a topical search (TS) was performed to find documents matching the search queries in Table 1 in the title, abstract, or keywords. In the query, an asterisk is used as a truncation symbol to find variations of the keywords. The query for food security was experimentally developed by Jayabalasingham et al. (2019), aiming to extract articles that closely match the wordings and intentions of the UN SDGs (United Nations, 2015). Several queries were formulated iteratively. For each iteration, the authors assessed how the top-cited articles were related to the food security goal, until reaching the query with the most satisfactory results. However, as IoT is a coined term, articles were retrieved by setting the full and abbreviated writing as the query. Data were retrieved on December 16, 2020.

**Table 1:** Search queries for extracting academic articles on food security and IoT.

**Query**	**Articles**
TS = (“food security” OR “food insecurity” OR “food production” OR “food productivity” OR “agricultural production” OR “agricultural productivity” OR “agricultural practices” OR “agricultural management” OR malnourish* OR malnutrition OR undernourish* OR “undernutrition” OR “land tenure rights” OR (smallholder AND (farm OR forestry OR pastoral OR agriculture OR fishery OR “food producer” OR “food producers”)) OR “land right” OR “land rights” OR “land reform” OR “land reforms” OR “resilient agricultural practices” OR “food nutrition improvement” OR “hidden hunger” OR “genetically modified food” OR (“gmo” AND food) OR “agroforestry practices” OR “agroforestry management” OR “agricultural innovation” OR (“food security” AND “genetic diversity”) OR (“food market” AND (restriction OR tariff OR access OR “north south divide” OR “development governance”)) OR “food governance” OR “food supply chain” OR “food value chain” OR “food commodity market”) NOT TS = (“disease”)Timespan: All years.	99,881
TS = (“Internet of Things” OR “iot”);Timespan: All years.	61,462

### Step 2

Core literature for both topics were cleaned and extracted. In order to increase the accuracy of the datasets, unrelated articles with keywords within the search query were excluded. This study was premised on the idea that academic literature does not exist in isolation, and a given article was expected to cite or be cited by another article on the same topic. This was captured through citation networks. A citation network was created, with each article represented as a node connected to other nodes in the list of references in the data sets. This type of direct citation network provides better topical representations (Klavans and Boyack, 2017). The largest connected component of each network was retained, while disconnected nodes (articles) were excluded.

### Step 3

As the network structure for the core literature was known, a network clustering algorithm was applied to identify tightly connected groups of articles within each topic. A modularity maximization algorithm was applied to measure how well a network was divided by comparing the strength of inter-cluster vs. intra-cluster connections. Modularity *Q* was defined in Eq. 1:

(1) $$\begin{array}{l} Q=\;\underset{i}{\sum }\left(e_{ii}-\;a_{i}^{2}\right),\;a_{i}=\underset{j}{\sum }e_{ij}\; \end{array}$$

where *e*_*ij*_ was the fraction of edges connecting *cluster i* and *cluster j*, while *e*_*ii*_ was the fraction of edges within *cluster i*. There are several algorithms for extracting clusters based on modularity maximization that differ in the number and characteristics of clusters produced, computational speed, and suitability for large networks. This study used the Louvain algorithm (Blondel et al., 2008), as it has been applied to citation networks in a variety of topics and is known to scale well in large networks (Šubelj et al., 2015). It also produces fewer clusters, which ease the interpretation of large network trends compared to other modularity maximization algorithms that produce a mix of few large clusters and many small clusters (Dao et al., 2019). For guidance in understanding major trends represented by clusters, they were named based on a manual inspection of contents of the clusters' most cited articles. These clusters represent an academic landscape or the main subtopics for food security and IoT.

These main clusters were further subclustered to obtain fine granular topics containing more specific vocabularies. The sub-clusters had two different purposes. First, they allowed literature to be split into fine-grained clusters and avoid the problem of resolution limits (Fortunato and Barthélemy, 2007) found in large networks. As their size decreases, they become more cohesive and easier to interpret. Specifically, their vocabulary was expected to be narrower. Second, more subclusters allow for more pairwise comparisons between subclusters of both networks. This number of intersections is the mechanism that helps create a network of co-occurring B-terms.

### Step 4

Papers shared by both topics were excluded. Intersecting articles were checked separately to extract available knowledge regarding the intersection of both topics and contrast the findings to assess similarities and differences.

### Step 5

Semantic similarities of each subcluster were computed and B-terms for each pair of subclusters were extracted. First, text was prepared by concatenating keywords, abstracts, and titles of each article, lowercased, with stop words removed, and stems of each word obtained. Following this, each article was represented as a vector whose length was the size of the vocabulary present in the dataset, with values being the number of occurrences of each word in the article. Text vectors at the cluster level were obtained by the summation of the text vectors of each article in the cluster. Finally, values were transformed into *tfidf* weights, as follows:

(2) $$\begin{array}{l} w_{c}^{\left(i\right)}=tf_{i,c}•log\left(\frac{N}{df_{i}}\right),\; \end{array}$$

where *tf*_*i,c*_ was the frequency of term *i* in subcluster *c*, *df*_*i*_ was the number of documents with *i*, *N* was the total number of documents, and *w*_*c*_ was normalized such that ||*w*_*c*_|| = 1. A similarity score was computed between all possible pairs of subclusters and B-terms extracted from the pairs that were above average. There were a variety of similar scores applied to text vectors, and the selection of one could affect the results. In order to assess the robustness of selecting any of them, correlation of four similarity scores was computer. A high correlation indicates that a similar result can be obtained regardless of the metric. The following similarity scores were compared:

(3) $$\begin{array}{l} Cosine\left(c_{1},c_{2}\right)=w_{c_{1}}•\;w_{c_{2}} \end{array}$$

(4) $$\begin{array}{l} Jaccard\left(c_{1},c_{2}\right)=\frac{w_{c_{1}}•\;w_{c_{2}}}{\underset{i}{\sum }w_{c_{1}}^{2}+\underset{i}{\sum }w_{c_{2}}^{2}-\;w_{c_{1}}•\;w_{c_{2}}}\; \end{array}$$

(5) $$\begin{array}{l} Dice\left(c_{1},c_{2}\right)=\frac{2\;\left(w_{c_{1}}•\;w_{c_{2}}\right)}{\underset{i}{\sum }w_{c_{1}}+\;\underset{i}{\sum }w_{c_{2}}\;}\; \end{array}$$

(6) $$\begin{array}{l} Simpson\left(c_{1},c_{2}\right)=\frac{\;|w_{c_{1}}•\;w_{c_{2}}|}{min\left(\left|w_{c_{1}}|,|w_{c_{2}}\right|\right)\;} \end{array}$$

where *w*_*c*_ was the *tfidf* vectors computed with Equation (2).

### Step 6

A network of B-terms was created. B-terms were connected if they appeared together as intersecting terms between a pair of subclusters. The more frequently they appeared together, the stronger their connection. As in citation networks, clusters of B-terms rather than articles were obtained by applying the Louvain algorithm. Clusters of B-terms were expected to share semantic similarity or belong to the same topic. Each cluster of B terms was assigned a name based on the list of terms within the cluster. The result was the network and list of B-terms that conformed to it. A few LBD methods rely on visualizations of B-terms (Cohen et al., 2010; Goodwin et al., 2012; Workman et al., 2016; Henry et al., 2019). However, they were dependent on the use of biomedical controlled vocabularies; hence, this approach relied on standard network visualization techniques. To conclude, results were compared with other LBD methods to identify similarities, differences, and use cases for each.

### Implementation

The method described in steps 2 to 6 was implemented using the programming language R version 3.6.3 (R Core Team, 2019). Additionally, network building and clustering were performed using the R package, Igraph version 1.2.5 (Csardi and Nepusz, 2006). The network of academic articles in step 4 was plotted using the Large Graph Layout algorithm and free software designed to plot large networks (Adai et al., 2004). The size of nodes was set to zero to display only the edges. These were given different colors to represent clusters. Finally, for the network of B-terms described in step 6, VOSviewer (Van Eck and Waltman, 2010) and Gephi (Bastian et al., 2009) were used, as these were considered to be an appropriate option for smaller networks. Gephi's OpenOrd layout with software default parameters was used to plot the network of B-terms.

## Results and Discussion

### Major Clusters on Food Security and IoT

**Figure 2:**
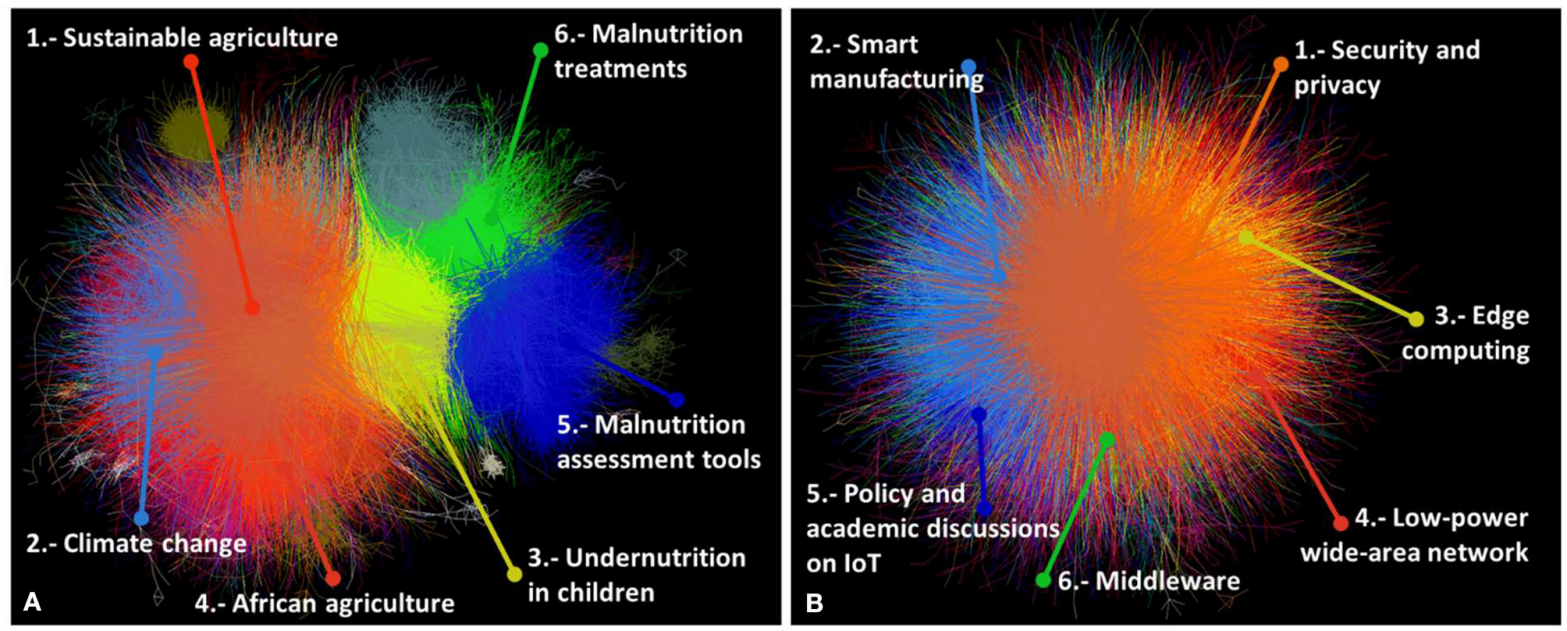
Citation network of **(A)** food security and **(B)** IoT research. The top six large clusters are shown.

Before exploring connecting terms, the academic landscape of food security and IoT was examined by looking at clusters derived from citation networks of academic articles on each topic. A total of 77,559 articles was found in the largest component of the food security network, which can be grouped into 17 major clusters. The 41,117 IoT research articles were divided into 16 clusters. Figure 2 shows the networks and relative positions of the largest clusters.

Research on food security has a long history, with the earliest article in the network being one on children malnutrition published in 1919 (Blanton, 1919). The IoT is a younger field of research, with the earliest article using the concept published in 2002 (Schoenberger, 2002). Details of major clusters are summarized in Tables 2, 3, respectively. Additionally, Table 4 shows the countries most engaged based on the number of publications on food security.

**Table 2:** Food security clusters.

**ID**	**Cluster**	**Articles**	**Avg. year**	**Avg. citations**	Max. citations*
1	Sustainable agriculture	11,391	2014.1	30.0	3891
2	Climate change	8,579	2014.9	25.6	2731
3	Undernutrition in children	7,584	2011.4	20.4	1535
4	African agriculture	7,530	2013.8	16.5	546
5	Malnutrition assessment tools	7,518	2011.1	26.6	1856
6	Malnutrition treatments	5,391	1998.6	24.0	1708
7	Food insecurity	5,183	2015.1	16.9	473
8	Experimental testing of malnutrition	4,643	1999.9	26.7	972
9	Agricultural management systems	4,007	2012.9	29.6	1016
10	Land tenure and property rights	3,475	2012.0	16.8	731
11	Urban agriculture	2,316	2014.3	19.2	491
12	Plant breeding	2,003	2014.8	32.2	1604
13	Food supply chain	1,525	2015.5	19.6	544
14	Aquaculture	1,455	2015.6	22.0	436
15	Genetically modified food	977	2013.3	19.7	293
16	Wildlife and food security	650	2013.4	21.6	601
17	Other	3,332	2010.9	26.0	755

*In each cluster, citations range from zero to maximum.

**Table 3:** IoT clusters.

**ID**	**Cluster**	**Articles**	**Avg. year**	**Avg. citations**	Max citations*
1	Security and privacy	6,083	2018.0	8.3	648
2	Smart manufacturing	5,388	2017.5	11.7	1725
3	Edge computing	4,473	2018.6	10.1	1343
4	Low-power wide-area network	4,220	2018.3	8.1	1218
5	Policy and academic discussions on IoT	3,921	2016.7	8.4	6057
6	Middleware	3,130	2017.2	6.9	1125
7	Protocols and architectures	2,165	2017.8	7.7	2341
8	Blockchain and smart contracts	1,887	2018.8	9.9	965
9	Smart cities	1,693	2017.6	9.1	2058
10	Energy harvesting for IoT devices	1,452	2018.3	14.2	680
11	IoT for energy management	1,384	2018.1	7.1	466
12	IoT for healthcare	1,360	2018.1	7.4	780
13	Ambient backscatter	782	2018.1	8.9	409
14	Bluetooth	702	2017.9	6.7	398
15	Cognitive IoT	619	2018.3	8.7	236
16	Others	1,858	2017.8	6.7	300

*In each cluster, citations range from zero to a maximum.

**Table 4:** Countries with most publications on food security research.

**Cluster**	OECD status*	**Top five countries by number of publications**
1	OECD	USA (3,195); United Kingdom (1,319); Germany (1,134); France (763); Netherlands (748)
	Non-OECD	China (1,432); Brazil (680); India (469); Argentina (215); Indonesia (181)
2	OECD	USA (2,656); Australia (870); United Kingdom (813); Germany (722); Netherlands (431)
	Non-OECD	China (1,776); India (638); South Africa (297); Kenya (213); Pakistan (212)
3	OECD	USA (2,926); United Kingdom (1,073); Canada (390); Australia (377); Switzerland (300)
	Non-OECD	India (862); Bangladesh (486); Ethiopia (432); South Africa (355); Brazil (322)
4	OECD	USA (1,801); Netherlands (851); United Kingdom (792); Germany (674); Australia (480)
	Non-OECD	Kenya (938); Ethiopia (526); South Africa (415); China (407); Zimbabwe (380)
5	OECD	USA (1,608); United Kingdom (660); Japan (525); Australia (514); Italy (405)
	Non-OECD	China (383); Brazil (344); Taiwan (156); India (120); Iran (72)
6	OECD	USA (1,579); United Kingdom (611); France (337); Canada (242); Japan (171)
	Non-OECD	India (489); Brazil (238); Nigeria (166); Malawi (156); South Africa (154)
7	OECD	USA (3,080); Canada (694); United Kingdom (396); Australia (277); Italy (81)
	Non-OECD	South Africa (330); Ethiopia (122); Brazil (109); India (87); Iran (85)
8	OECD	USA (1,319); United Kingdom (547); France (258); Spain (206); Australia (204)
	Non-OECD	Brazil (617); India (207); China (176); Argentina (105); South Africa (47)
9	OECD	USA (1,081); United Kingdom (313); France (260); Germany (247); Canada (242)
	Non-OECD	China (1,000); India (154); Brazil (80); Iran (78); Pakistan (47)
10	OECD	USA (1,014); United Kingdom (438); Netherlands (233); Australia (221); Canada (207)
	Non-OECD	China (422); South Africa (303); Indonesia (82); Zimbabwe (69); India (53)
11	OECD	USA (607); United Kingdom (407); Australia (183); Germany (162); Canada (150)
	Non-OECD	South Africa (75); China (55); Brazil (37); India (34); Ghana (22)
12	OECD	USA (402); Australia (123); United Kingdom (116); Germany (97); Italy (77)
	Non-OECD	China (449); India (319); Pakistan (144); Brazil (82); Nigeria (49)
13	OECD	USA (273); United Kingdom (238); Italy (139); Netherlands (112); Spain (77)
	Non-OECD	China (167); India (90); Brazil (46); Malaysia (34); South Africa (27)
14	OECD	USA (420); Australia (286); United Kingdom (259); Canada (215); Germany (104)
	Non-OECD	China (91); Malaysia (73); Brazil (64); South Africa (62); Vietnam (57)
15	OECD	USA (328); United Kingdom (125); Germany (65); Canada (61); Italy (59)
	Non-OECD	China (80); India (17); Brazil (16); Kenya (13); South Africa (13)
16	OECD	USA (165); United Kingdom (101); Italy (54); Canada (39); Germany (34)
	Non-OECD	India (52); South Africa (42); Ethiopia (35); China (29); Indonesia (24)
17	OECD	USA (893); United Kingdom (292); Germany (254); Italy (215); Australia (206)
	Non-OECD	China (264); Brazil (118); India (93); South Africa (65); Kenya (60)

*Member of the Organization for Economic Co-operation and Development (OECD) as of 2021.

Based on average publication years, early research on food security focused on issues related to malnutrition treatment, while the latest trends focused on aquaculture and the food supply chain. However, plant breeding had the most impact in terms of average citations per paper, which collected research exploring techniques for enhancing the breeding of micronutrients in genetically modified food crops (Welch and Graham, 2004). Other studies explored the enhancement of salt tolerance in plants, improving agricultural productivity (Apse et al., 1999). The most cited article across this dataset was in the cluster of sustainable agriculture, which was a discussion of soil carbon sequestration, a technique of regenerative agriculture that could play a significant role in land restoration and slowing climate change (Lal, 2004). Sustainable agriculture was also the dominant cluster based on the number of publications.

One of the clusters focused on research from and for Africa, with a focus on techniques and policies affecting small farmers (Barrett, 2008; Pretty et al., 2011). Although the US and the UK led in food security research (Table 4), African countries participate across clusters of this topic. Other countries with active engagement in food security research were China, India, and Brazil.

IoT as a concept and subject of research is relatively new. The idea of devices connected to the Internet and ubiquitous computing date back to the 1980's (Weiser, 1991), while the term itself started circulating since 1999 (Ashton, 2009). However, a large volume of publications and developments in IoT have appeared recently. The dataset in the present study included a 2-year difference between the average publication year of articles in the oldest and newest clusters. The largest number of publications was related to security and privacy issues, followed by smart manufacturing. The cluster on energy harvesting for IoT devices captured more citations on average. The most cited article was a review discussing the paradigm of IoT (Atzori et al., 2010), which appeared in the cluster of policy and academic discussions on IoT.

### Intersecting Terms Between Food Security and IoT

Major clusters were split into subclusters, leading to 304 subclusters for food security and 304 for IoT. Similarity between all possible pairs of subclusters in the two networks was obtained and pairs above average were retained. As selection of similarity above others could result in different results, robustness of the approach was verified by comparing commonly used similarity metrics on text mining. Table 5 presents the correlations between them. There was an exact correlation between Cosine and Dice scores, while Jaccard and Simpson showed a high correlation. Therefore, the selection of one score above others had little to no impact on the results of the present study.

**Table 5:** Correlation between different similarity measures.

	**Cosine**	**Jaccard**	**Dice**	**Simpson**
Cosine	1.00000			
Jaccard	0.99220	1.00000		
Dice	1.00000	0.99220	1.00000	
Simpson	0.99935	0.99169	0.99935	1.00000

**Figure 3:**
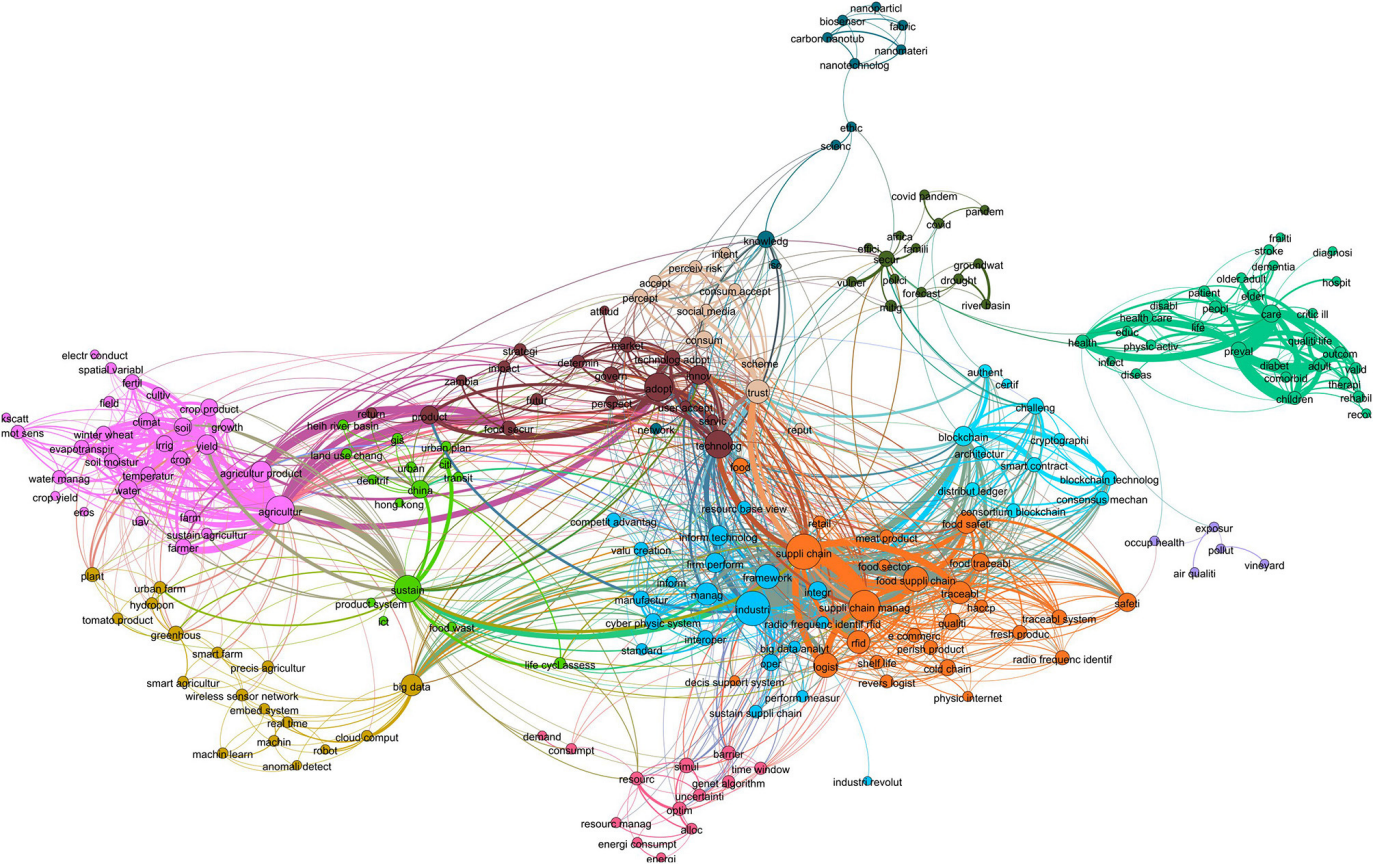
Cooccurrence network of B-terms between food security and IoT.

Cosine similarity is considered the standard for topic similarity in information retrieval (Manning et al., 2008), while it has been found to outperform other metrics in LBD research. For instance, Cosine similarity was found to be the best metric to establish connections between clusters of academic articles and patents sharing the same topic when metrics were evaluated against the opinions of experts (Shibata et al., 2011). Similarly, Cosine similarity showed the best content relatedness when comparing clusters of social issues and technologies in a set of academic articles (Ittipanuvat et al., 2014). Based on the results presented in Table 5 and previous research, Cosine similarity was selected to extract intersecting terms. Figure 3 shows the network of intersecting terms between a pair of subclusters of food security and IoT with an above-average similarity score.

Each term in the network was connected to another if they cooccurred between pairs of subclusters. The size of the nodes represented the degree or number of connections, and the thickness of the edges was the number of times the two terms cooccured. Terms that appeared two or more times are shown. There were 13 clusters of terms. For ease of exploration, each cluster was assigned a name that summarized each group of keywords as a whole. These clusters can be understood as transversal themes in common between food security and IoT, including healthcare, agriculture, supply chain, information technologies, social acceptance, machine learning, China, risk mitigation, resource management systems, blockchain, nanomaterials, perceived risks, and occupational health. Table 6 shows the summary statistics of articles containing non-unigram B-terms in the datasets of food security and IoT for the 13 themes.

**Table 6:** Transversal themes across food security and IoT.

**Theme**	**Food security**					**IoT**				
	**Articles**	**Articles %**	**Avg. year**	**Avg. citations**	**Max. citations**	**Articles**	**Articles %**	**Avg. year**	**Avg. citations**	**Max. citations**
Blockchain	23	0.03%	2020.0	12.7	65	412	1.00%	2019.0	15.8	965
Nanomaterials	15	0.02%	2016.7	29.1	233	48	0.12%	2018.3	30.0	262
Supply chain	1,569	2.02%	2015.5	20.6	612	810	1.97%	2017.6	18.5	1,725
Information technologies	139	0.18%	2015.7	20.2	294	1412	3.43%	2018.1	16.3	1,725
Machine learning	723	0.93%	2015.9	16.8	476	8758	21.30%	2018.1	11.8	6,057
Healthcare	2,365	3.05%	2014.3	20.8	590	457	1.11%	2018.4	15.3	1,725
Automation	406	0.52%	2016.4	16.8	185	17	0.04%	2019.6	9.4	68
China	2,425	3.13%	2015.6	28.7	2784	81	0.20%	2018.1	19.2	244
Technology adoption	8,579	11.06%	2015.7	20.4	3891	108	0.26%	2018.4	8.0	76
Consumer acceptance	188	0.24%	2015.3	26.0	361	78	0.19%	2018.1	10.7	107
Resource management systems	315	0.41%	2015.3	23.8	408	910	2.21%	2018.6	7.8	863
Agriculture	6027	7.77%	2014.1	26.1	3342	179	0.44%	2018.3	6.5	123
Air quality	66	0.09%	2014.2	29.8	699	132	0.32%	2018.6	4.5	65

In Table 6 themes were sorted by the difference in average citations for related papers in each dataset. Articles containing blockchain B-terms in the IoT dataset had the largest difference in citations compared with articles in food security. On the other hand, air quality had more citations in the food security dataset than in IoT, revealing a gradient of themes in which academics tend to emphasize it as a solution (at the top) or concern (at the bottom). Relationships were found by exploring the content of articles on such themes. Out of the 13 themes, this paper focused on three practical examples.

The cluster composed of B-terms related to the blockchain and smart contracts pointed to the application of distributed ledger technologies to transfer and manage assets in the context of the food security loan program in China (Wang et al., 2019). This kind of application was incipient in the context of food security; however, its underlying technology could be enhanced by many developments linking the blockchain and IoT.

The intersecting theme of air quality matched research on urban agriculture and IoT monitoring systems. For instance, the need to monitor heavy metal content in food due to polluted air in urban areas (Ercilla-Montserrat et al., 2018) could be satisfied with a system of sensors transmitting through a wide-area low-power network (Zheng et al., 2016). In addition, crop production has been found to greatly benefit from air quality control systems. However, the implementation of such systems requires globally orchestrated efforts or incentives from local governments (Shindell et al., 2012). Current developments in IoT for air quality monitoring could help bridge the gap between understanding and practice.

Finally, the last category, labeled China, appeared due to case studies covering Chinese research focused on IoT devices for agriculture and the service sector in rural China. This theme differed from others in that it focused on a geographic location. From the perspective of public knowledge discovery, China offered a variety of examples for potential applications of IoT in the context of food security, from which policymakers and researchers could draw ideas for implementation in other locations. For instance, this category included remote monitoring systems for agriculture (Zhang et al., 2017) and a management system for water conservation (Liu et al., 2015) developed in rural China.

### Comparison With Other LBD Methodologies

The network presented in Figure 3 is a way to visualize B-terms that eases the exploration of long lists of terms by grouping them into semantically related keywords. Currently, there are other LBD methods available, most of which have been developed for biomedical literature but are generalizable to some extent. Table 7 presents the B-terms obtained by applying three LBD methods to the data in this study. First, B-terms were obtained by applying the method of Swanson and Smalheiser (1997), which is the basic implementation of LBD for closed discovery. Second, queries from Table 1 were applied to the Arrowsmith web service (Smalheiser et al., 2009). Only bigrams were retained to avoid a long list of generic unigrams. Arrowsmith pulls data from Medline, favoring biomedical literature and ranking newer terms higher. This makes surface COVID-19 related terms highly ranked. Finally, results of the method of Ittipanuvat et al. (2014) were demonstrated. This method ranks the intersecting terms based on pairs of clusters with high similarity. The Ittipanuvat et al. method generates *n* ^*^ *m* lists of B-terms, where *n* and *m* are the number of clusters for literature A and C, respectively. This study obtained 272 lists of B-terms (16 × 17 clusters), one per pair of clusters. Table 7 shows the resulting B-terms for three most semantically similar clusters. LBD using the Ittipanuvat et al. method requires more intentionality from the researcher by having a research question related to the pair of clusters or a methodological approach to filter the pairs of clusters for analysis. Additionally, each list of B-terms has the same characteristics as the Swanson and Smalheiser methods. Hence, when no controlled vocabulary is available, each B-term must be considered.

**Table 7:** B-terms between food security and IoT by using other LBD methods.

***Method***	***B-terms***
Swanson and Smalheiser (1997)	Blockchain; trust; supply chain; traceable; supply chain management; elder; older adult; blockchain technology; food supply chain; smart contract; hospital; adult; value creation; critic ill; hypertension; dementia; blood pressure; reverse logistics; carbon.
Arrowsmith	Mobile phone; sedentary behavior; ionic liquid; silver nanoparticle; antiretroviral therapy covid-19 pandemic; scoping review; vegetation index; smart city; molecularly imprinted big data; leaf nitrogen; bariatric surgery; metal-organic framework; glycemic control.
Ittipanuvat et al. (2014)	“*Food supply chain” and “Smart manufacturing”:* Safety; risk; monitoring; food; existing; information; environmental; data; consumer; field; market; environment “*Food supply chain” and “Blockchain and smart contracts”:* Safety; risk; monitoring; food; concern; single; existing; information; data; market; environment “*Malnutrition assessment tools” and “IoT for healthcare”:* Detection; real time; safety; real; product; method; consumer; time; identification; risk; quality; system; processing; technology; control; standard; health; monitoring; developed; information; assessment; approach; network; different; performance; model; process; platform; IoT.

Similarities were found between the proposed method and other known LBD approaches. Keywords such as healthcare, supply chain, and blockchain were observed with the classic ABC approach, while other terms, such as climate change, were missing in this network. On the other hand, keywords related to agriculture, nanomaterials, and air quality surfaced in the network. Previous methodologies reported extensive lists of connecting terms, putting the burden of assessment of each keyword on the reader or expert. Although the present method is not different in this regard, semantic grouping of keywords were used to ease the assessment process, for instance, by disregarding entire clusters of irrelevant keywords. This advantage is only apparent when the method is applied to non-biomedical fields. Biomedicine tools, such as Arrowsmith, can take advantage of semantic categories already in place in the Medline Subject Heading (MESH) thesaurus to group keywords into different fields.

To identify relevant connecting keywords, the transversal theme knowledge domain is required. The method proposed in this study, like others, is prone to revealing generic terms, such as product, technology, and future (Figure 3). However, as the keywords are grouped in clusters, they facilitate finding concepts that might bring interesting connections between studies and ease the discovery process.

Comparing different methods of LBD has been a challenge due to the lack of evaluative frameworks and ground truth (Smalheiser, 2012). In biomedical LBD, some evaluation attempts were performed by replicating the foundational discoveries of Swanson between Fish Oil and Raynaud's Syndrome and relations between Migraine and Magnesium (Sebastian et al., 2017). There are no similar comparative datasets in social sciences. Rather, different non-biomedical LBD methods may serve different purposes. The method applied in this study served as an exploratory tool to identify transversal themes between two research topics. The methods of Swanson and Smalheiser and Ittipanuvat et al. can help to explore linking concepts for specific and broad topics, respectively. According to these approaches, a theme refers to a group of concepts or terms; hence, it is more an aggregated exploratory level.

### Articles Intersecting Food Security and IoT

A total of 74 common documents were found between the largest components of both networks. Most of them were distributed in clusters of smart manufacturing, wide-area low-power networks, and blockchains and smart contracts in the IoT dataset. On the other hand, they were concentrated in the food supply chain in the food security dataset. These documents represent the current direct linkage between these topics, where the potentialities of the IoT for helping with food security issues are already acknowledged.

Research in this set of documents included the development of value-centric frameworks of the IoT for the food supply chain, with a paradigm shift from traceability values of IoT systems to income values that include shelf life prediction, sales premium, precision food production, and insurance cost reduction (Pang et al., 2015). Other research has focused on showcasing systems for smart farms (Muangprathub et al., 2019) or applications of the blockchain in the food supply chain (Zhao et al., 2019). While these were direct connections between the topics of this study, the approach applied in this paper reveals interesting terms or indirect possible connections that became visible after removing common documents from the network. A list of 74 documents is included in the Supplementary Material.

### Poverty Alleviation and Food Security

The UN SDGs agenda places the end of poverty and zero hunger as the first and second goals (United Nations, 2015). Although separate topics, research targeting one may have spillover effects into the other. This section explores transversal topics between food security and poverty alleviation in order to understand themes that may be expected to address issues of SDGs. Research on SDGs was triangulated by applying the methods discussed above and using poverty alleviation data from previous research (Mejia and Kajikawa, 2020).

**Figure 4:**
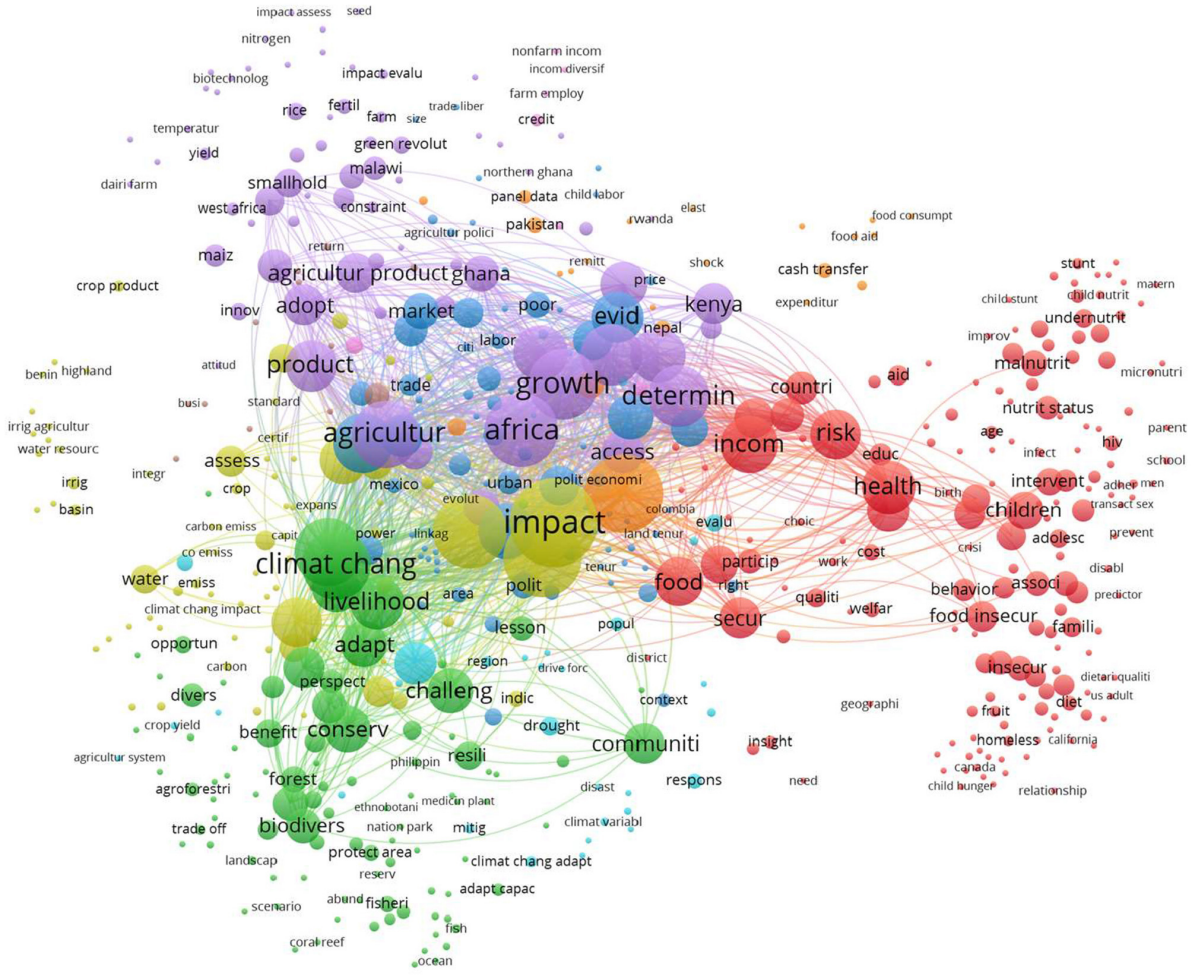
Cooccurrence network of B-terms in food security and poverty alleviation.

There was a larger overlap between both topics, with 1,253 articles simultaneously tackling poverty alleviation and food security. These were removed from the networks, and B-terms were computed based on the remaining articles. Figure 4 shows the network of B-terms.

The network was composed of 567 B-terms aggregated into nine clusters. Compared to that of food security and IoT, where there were 271 B-terms aggregated into 13 clusters, this network was more intertwined, which can be interpreted as being more topically overlapped. After checking the keywords and articles related to the B-terms, nine themes were identified: welfare, sustainability, economic development, China, Africa, disaster management, migration, agriculture, and income generation. These themes had a broad scope, with two empathizing research in China and Africa.

Therefore, developing policies that consider linkages between a social challenge and a technological solution, such as between food security and IoT, can have an extended benefit in solving other social pressures while simultaneously targeting any broad themes shared by social issues. The LBD method helps reveal themes with this added value.

## Conclusion and Limitations

This study applied an LBD methodology to find intersecting terms between disjoint research literature. The network approach helped visualize and organize terms into semantically-related clusters of keywords, which could help experts navigate a potentially long list of terms. As such, transversal themes were revealed and a taxonomy was automatically created.

This method was applied to the topics of food security and IoT, representing a variety of social issues and technologies, respectively. At the current state of research, both topics were highly disjoint. Only 74 (0.06%) out of 118,602 articles in the networks appeared in both. This indicates opportunities for researchers to dig deeper and establish direct connections, devices, or strategies for IoT to help solve this SDG. However, recognizing this gap is insufficient to set directions for researchers or policymakers. LBD methods help provide potential solutions that can be further investigated in research laboratories. The network of B-terms is intended to help identify possible connections. This study found 13 transversal themes between food security and IoT. Each theme was a cluster of keywords derived from multiple connections between the subclusters of the two topics.

Finding transversalities between social issues and potential technological solutions is part of evidence-based policymaking, which pursues two goals: “to use what we already know from program evaluation to make policy decisions, and to build more knowledge to better inform future decisions” (Evidence-Based Policymaking Collaborative, 2016). Rigorous research is expected to provide evidence for policy debates and internal public sector processes for improving program development and reforms (Head, 2009). The present LBD method helps navigate academic literature in order to compile evidence on what has worked, under what conditions, and when as well as the cost and benefits of implementing these solutions. In practice, policymakers and other stakeholders interested in the application of IoT in the context of food security could use the provided themes and linked literature to narrow down the pool of potential options and classify various alternatives. Additionally, the map could help to bring completeness to policy proposals by finding themes not yet covered. The applied method does not establish a direct link but helps rethink IoT policies and technology roadmaps in order to address pressing social issues, such as food security.

There was active participation in academic publishing on food security from non-OECD countries in Africa and Asia, which have an identified demand for information on potential ways to attain sustainable agriculture. Technological interventions have been applied by trial and error, which did not always consider regional differences (Giller et al., 2011). Additionally, some policy reforms on land use and agriculture had a positive impact on government budgets but an unintended negative impact on food security in rural areas in Africa (Morris et al., 2007). Failure in this kind of policy signals the need for supportive tools to identify potentially better options for application (Denning et al., 2009). In Asia, China and India engage in research on IoT and food security, although the efforts seem to be rated in parallel rather than conjoined.

While this paper illustrated the application of an LBD methodology to support policy discovery, it is limited in being an exploratory tool scoping technological alternatives. Achieving food security is influenced by several factors, such as social capital, strength of rural institutions, access to credit, and markets (Teklewold et al., 2013). However, the present study contributed by illustrating a case in which a social issue can be addressed by encouraging innovation and supporting evidence-based policymaking.

The methods used in this study had some limitations. There was a lack of an evaluation framework to assess the quality of the terms and clusters obtained and the impact of potential discoveries. This is a common problem for most LBD methods, given the non-existence of a standard for comparison and the costs of testing multiple hypotheses created by exploring connecting terms (Smalheiser, 2012; Sebastian, Y., et al., 2017). In addition, although the method is assumed to be generalizable, more case studies are required to understand its applicability in other fields.

Some opportunities for future improvement include the application of other clustering algorithms, such as trajectory clustering, which can, to some extent, be transferred to the dynamics of citation networks (Yuan et al., 2017; Bian et al., 2018). The application of LBD to datasets different from academic articles is also a promising new avenue of research. Social challenges may first be identified in other media, such as news, tweets, or policy reports. For instance, the Global Data on Events, Locations, and Tone (GDELT) dataset offers large-scale monitoring of social events based on mining web sources (Leetaru and Schrodt, 2013), which may be suitable to track global issues related to the SDGs, as in this paper.

This study offered a method for exploring shared terminologies between disjoint literature that does not depend on controlled vocabularies by reducing the burden of policymakers or experts aiming to develop a hypothesis or reframe a policy strategy. In addition, this study provided more use cases of LBD beyond the biomedical fields. Future research should focus on developing evaluation systems for these outputs, methodological improvements, and ease of accessibility through programming libraries or web interfaces.

## Data Availability Statement

The data analyzed in this study is subject to the following licenses/restrictions: A Web of Science license is needed to download the data as instructed in the article. Post-processed datasets for the reproduction of the network and intersecting terms are available upon request. Requests to access these datasets should be directed to mejia.c.aa@m.titech.ac.jp.

## Author Contributions

CM designed the project, collected the data, and conducted the analysis. YK supervised and monitored the project. All authors made substantial, direct, and intellectual contributions to the study.

## Conflict of Interest

The authors declare that the research was conducted in the absence of any commercial or financial relationships that could be construed as a potential conflict of interest.
